# Comparative Proteomic Analysis of Glycolytic and Oxidative Muscle in Pigs

**DOI:** 10.3390/genes14020361

**Published:** 2023-01-30

**Authors:** Xiaofan Tan, Yu He, Yuqiao He, Zhiwei Yan, Jing Chen, Ruixue Zhao, Xin Sui, Lei Zhang, Xuehai Du, David M. Irwin, Shuyi Zhang, Bojiang Li

**Affiliations:** 1 College of Animal Science and Veterinary Medicine, Shenyang Agricultural University, Shenyang 110866, China; 2 Liaoning Provincial Animal Husbandry Development Center, Liaoning Province Agricultural Development Service Center, Shenyang 110032, China; 3 Department of Laboratory Medicine and Pathobiology, University of Toronto, Toronto, ON M5S 1A8, Canada

**Keywords:** pig, glycolytic muscle, oxidative muscle, differentially expressed proteins, meat quality

## Abstract

The quality of meat is highly correlated with muscle fiber type. However, the mechanisms via which proteins regulate muscle fiber types in pigs are not entirely understood. In the current study, we have performed proteomic profiling of fast/glycolytic biceps femoris (BF) and slow/oxidative soleus (SOL) muscles and identified several candidate differential proteins among these. We performed proteomic analyses based on tandem mass tags (TMTs) and identified a total of 26,228 peptides corresponding to 2667 proteins among the BF and SOL muscle samples. Among these, we found 204 differentially expressed proteins (DEPs) between BF and SOL muscle, with 56 up-regulated and 148 down-regulated DEPs in SOL muscle samples. KEGG and GO enrichment analyses of the DEPs revealed that the DEPs are involved in some GO terms (e.g., actin cytoskeleton, myosin complex, and cytoskeletal parts) and signaling pathways (PI3K-Akt and NF-kappa B signaling pathways) that influence muscle fiber type. A regulatory network of protein–protein interaction (PPI) between these DEPs that regulates muscle fiber types was constructed, which demonstrates how three down-regulated DEPs, including PFKM, GAPDH, and PKM, interact with other proteins to potentially control the glycolytic process. This study offers a new understanding of the molecular mechanisms in glycolytic and oxidative muscles as well as a novel approach for enhancing meat quality by transforming the type of muscle fibers in pigs.

## 1. Introduction

Muscle fibers make up 75%–90% of the volume of skeletal muscle, which is a heterogeneous tissue. Thus, fiber type is the main factor defining muscle morphology [1]. Muscle fibers are classified into type I, Ⅱa, Ⅱb, and Ⅱx, based on their predominant myosin heavy chain (MyHC) isoforms [2,3]. In addition, muscle fibers are divided into oxidative (slow-twitch) and glycolytic (fast-twitch) fibers, depending on their contractile and metabolic characteristics [4]. Meat quality is a crucial economic feature in the livestock business. In recent years, with improving standards of living, consumer requirements for meat quality have constantly increased. According to prior studies, the type of skeletal muscle fiber and its metabolic characteristics are strongly associated with the quality of meat [5,6,7]. Metabolism in meat changes after slaughter, and it is affected by the muscle fiber type, thus affecting the quality of the meat [5,8]. Several studies have demonstrated that different muscle fiber types determine meat quality, including pH, color, tenderness, drip loss, and intramuscular fat content [9,10,11,12,13,14]. For example in pigs, drip loss in the longissimus dorsi muscle is negatively correlated with the proportions of type I and IIa muscle fibers, whereas it is positively correlated with type IIb fibers [10]. Increasing the proportion of glycolytic muscle fibers can accelerate the decline of post-slaughter pH in pig meat [13]. Therefore, a better understanding of the mechanisms regulating muscle fiber types can contribute to improving meat quality.

Numerous factors, including the breed, nutrition, hormones, and the levels of protein-coding and non-coding RNA gene expression, affect the type of muscle fiber. For example, expression levels of MYH3 can affect the composition of muscle fiber types in the muscles [15]. Several studies have suggested that miRNAs and circRNAs could be involved in the transformation of muscle fiber types [16,17]. Recent developments in proteomic technologies have led to their widespread use in the identification of proteins associated with meat quality in pigs, cattle, sheep, and other animal species [18,19,20,21,22]. Poleti et al. used a lab-free proteome to identify 164 proteins that were differentially expressed in tissues with high and low intramuscular fat content in Nelore cattle [18]. Recently, Song et al., using a DIA-based quantitative proteomic analysis, evaluated differentially expressed proteins in pork that was cooked by different methods [23]. Proteins that are candidate markers for pork quality using proteomic techniques, such as biomarkers for intramuscular fat and drip loss, have been identified previously [19,24,25]. A few studies have also examined the proteomic profile of pork muscle involving different fiber types.

Our previous research has demonstrated that soleus (SOL) has better meat quality than biceps femoris (BF) [26]. In this study, we selected typical glycolytic muscles (BF, biceps femoris) and oxidative muscles (SOL, soleus) that have different predominant muscle fiber types for a protein profile analysis using TMT-tagged proteomics. Through this analysis, we identified 204 differentially expressed proteins (DEPs) among the BF and SOL muscle tissue. Gene Ontology (GO) and Kyoto Encyclopedia of Genes and Genomes (KEGG) enrichment analyses of these DEPs revealed distinct cytoskeletal components and signaling pathways enriched in these two muscle types. Finally, we constructed a protein–protein interaction (PPI) network of the DEPs, through which potential key proteins involved in the interconversion of muscle fiber types were identified. Furthermore, several proteins and regulatory networks were discovered in this study; they vary between glycolytic and oxidative muscles and serve as candidate targets for modification to improve pork quality through muscle fiber type conversion.

## 2. Materials and Methods

### 2.1. Animals and Sample Collection

The biceps femoris (BF) and soleus (SOL) muscles of three male progeny descended from a cross between a Duroc boar and a Meishan sow served as the source of the muscle samples used in the investigations here. BF and SOL muscle sample collection positions and procedures were carried out as described in a previous study [27]. All the animals used in the experiments resided in identical environmental conditions before slaughter, all were slaughtered at 180 days of age (106.17 ± 0.76 kg; n = 3), and each tissue sample was taken for protein extraction in triplicate. Samples were collected immediately after slaughter and stored in liquid nitrogen; all the samples were preserved at −80 °C for further use. Ethical approval for experiments involving animals was obtained from the Institutional Animal Care and Use Committee of Shenyang Agricultural University (permit number 202006032).

### 2.2. Extraction and Quantification of Protein

Extraction of total protein from the muscle samples was performed as described previously [28]. PASP lysis buffer (8 M Urea, 100 mM NH_4_HCO_3_, pH 8) was used to lyse the samples followed by ultrasonication on ice for 5 min. The lysate was centrifuged for 15 min at a rate of 12,000× *g* and 4 °C. The supernatant was first reduced with 10 mM DL-Dithiothreitol (DTT) at 56 °C for 1 h and then alkylated with iodoacetamide (IAM) at room temperature in the darkness for 1 h. After that, the samples were resuspended in precooled acetone (4 times the volume of the samples), incubated for a minimum of 2 h at −20 °C, and centrifuged for 15 min at a rate of 12,000× *g* and 4 °C to obtain the precipitate. The precipitate was first resuspended in 1 mL of pre-cooled acetone and then centrifuged for 15 min at 12,000× *g* and 4 °C. The resulting pellet was dissolved in dissolution buffer (100 mM TEAB, 8 M Urea, pH 8.5).

Protein quantification was performed with a Bradford protein quantitative kit (Beyotime Biotechnology, Shanghai, China), following the manufacturer’s guidelines. Protein integrity assays were performed by subjecting 20 µg of each protein sample to SDS–PAGE (12%) for 20 min at 80 V, and 50 min at 120 V. The gel was visualized after staining it with coomassie brilliant blue R-250. All samples were satisfactory for protein integrity and were allowed to proceed to subsequent experiments.

### 2.3. TMT Labeling of Peptides

TMT labeling of peptides was performed as described previously [29]. Trypsin was added to each protein sample in 100 mM TEAB buffer and incubated for 4 h at 37 °C. Trypsin and CaCl_2_ were added to each of the samples, and digestion was continued overnight. To lower the pH value to below 3, formic acid was added, and the mixture was centrifuged at a rate of 12,000× *g* at room temperature for 5 min. The obtained supernatant was loaded on a C18 desalting column. The column was washed with washing buffer (3% acetonitrile and 0.1% formic acid) three times. The peptides were then eluted with elution buffer (70% acetonitrile and 0.1% formic acid). The eluents were lyophilized, resuspended in 100 μL of 0.1 M TEAB buffer, added to the TMT-labeling reagent (in acetonitrile), and incubated at room temperature for 2 h. The reactions were terminated by adding 8% ammonia solution.

### 2.4. LC-MS/MS Analysis

LC–MS/MS analysis was conducted with the aid of an EASY-nLC^TM^ 1200 UHPLC system (ThermoFisher, Waltham, MA, USA) coupled with a Q Exactive^TM^ HF-X mass spectrometer (ThermoFisher, Waltham, MA, USA) operating in the data-dependent acquisition (DDA) mode. A C18 Nano-Trap column (3 μm, 4.5 cm × 75 μm) was injected with a sample and then subjected to peptide separation in an analytical column (1.9 μm, 15 cm × 150 μm) with linear gradient elution. Survey scans were performed using a Q Exactive^TM^ HF-X mass spectrometer with a range of *m*/*z* 350–1500, a resolution of 60,000, an automatic gain control (AGC) target value of 3 × 10^6^, and a maximum injection time of 20 ms. Higher-energy collisional dissociation (HCD) spectra were obtained from the top 40 abundant ions with a resolution of 30,000, AGC target value of 5 × 10^4^, maximum injection time of 54 ms, normalized collision energy of 32%, intensity threshold of 1.2 × 10^5^, and dynamic exclusion parameter—20 s.

### 2.5. Protein Identification, Quantification, and Differential Expression Analysis

Peptide sequences were searched against the pig UniProt proteome database using Proteome Discoverer 2.4 software (Thermo Fisher Scientific, Waltham, MA, USA). Following are the search parameters used: enzyme trypsin; mass tolerance of precursor ion—10 ppm; mass tolerance of product ion—0.02 Da; carbamidomethyl cysteine—fixed modifications; oxidation of methionine and TMT plex—dynamic modification; acetylation, TMT plex, met-loss, and met-loss+acetyl—N-terminal modification. A maximum of two missed cleavage sites were allowed. Peptide spectrum matches (PSMs) with >99% confidence were considered as identified PSMs, and proteins containing ≥1 unique peptide were considered as identified proteins. The identified PSMs and proteins with FDR no more than 1.0% were taken and analyzed further. The difference in protein abundance between the BF and SOL groups was examined using a *t*-test, and proteins with fold change >1.2 or <0.83 and *p* < 0.05 were considered to be differentially expressed proteins (DEPs).

### 2.6. The Functional Analysis of Identified Protein

Interproscan software (version 5.22-61.0) was used for GO, and InterPro protein domain (IPR) functional annotation was performed through the Pfam database [30]. BlastP (version 2.2.26) was used for KEGG and COG (Cluster of Orthologous Groups of proteins) functional annotation with e ≤ 1 × 10^−4^.

### 2.7. GO and KEGG Enrichment Analysis

For GO and KEGG enrichment analyses, a hypergeometric test for significance was conducted. Significant enrichment was considered when GO and KEGG had a *p* value of less than 0.05.

### 2.8. Protein–Protein Interactions (PPI) Analysis

Protein–protein interaction networks were constructed and visualized using the STRING database (http://STRING.embl.de/ accessed on 25 November 2022) with default parameters and settings [31].

## 3. Results

### 3.1. Overview and Characteristic Analysis of the Proteomes

**Figure 1:**
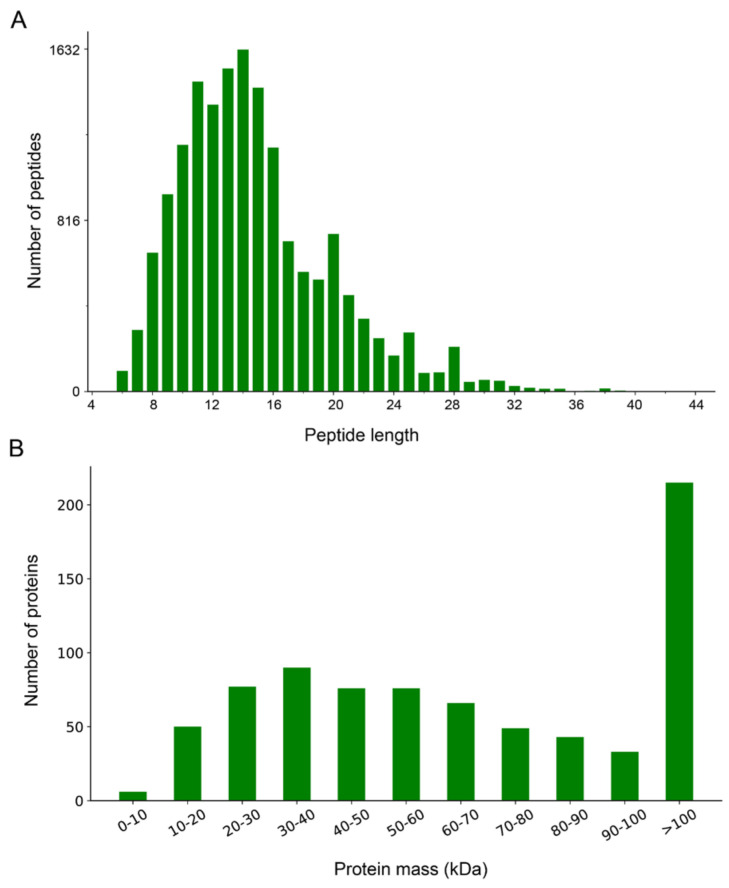
Overview and characterization of proteomes from BF and SOL muscles. (**A**) Distribution of length of the identified peptides. The X–axis represents peptide length, while the Y–axis represents the number of peptides. (**B**) Distribution of molecular weight of the identified proteins. The molecular weight of the proteins is represented by the X–axis, and the number of proteins is presented on the Y–axis.

To analyze protein differences between glycolytic and oxidative muscles, we constructed proteomic profiles based on BF and SOL muscle tissue samples using TMT-labeled quantitative proteomics. Overall, 26,228 peptides were identified in this study, which corresponds to 2667 proteins. Additionally, 2646 total proteins were quantified from the six muscle tissue samples (Table S1). Characterization of these identified peptides revealed that the peptide length ranged mostly between 7 and 25 amino acids, with 14-amino-acid-long peptides being the most abundant (Figure 1A). These results are consistent with data from subcutaneous fat in pigs [25]. In addition, molecular weight analysis revealed that most of the identified proteins had a weight range of 10–60 kDa, although a few were greater than 100 kDa (Figure 1B).

### 3.2. Functional Annotation of the Proteins

**Figure 2:**
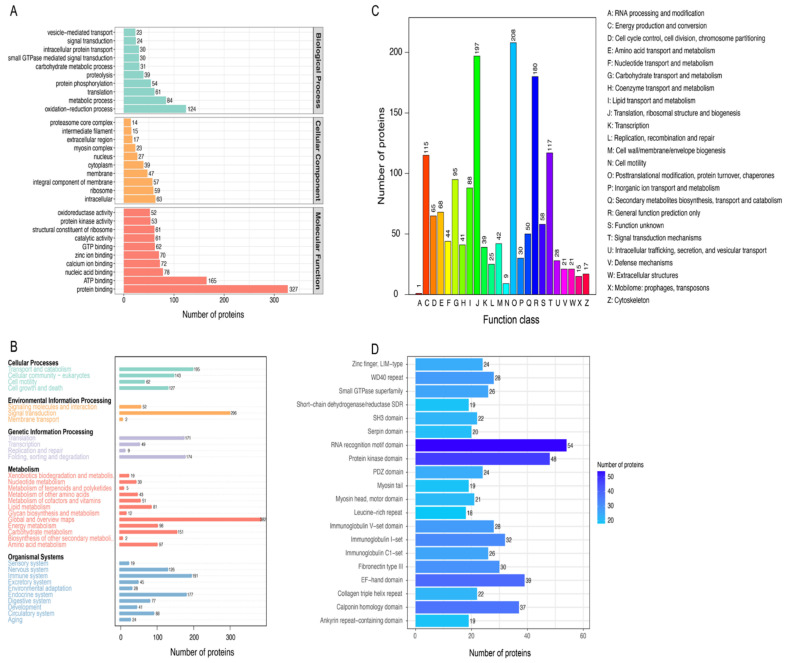
Functional annotation of the identified proteins. (**A**) GO annotation of the identified proteins. The X–axis represents the number of proteins, and the Y–axis represents the name of the GO term. (**B**) KEGG annotation of the identified proteins. The X–axis displays the number of proteins, while the Y–axis represents the name of the pathway. (**C**) COG annotation of the identified proteins. The X–axis represents the functional classification in the COG database, and the Y–axis represents the number of proteins. (**D**) IPR annotation of the identified proteins. The X–axis displays the number of proteins, while the Y–axis displays the name of the protein domain.

To explore the potential functions of the identified muscle proteins, we performed GO, KEGG, COG, and IPR functional annotations. The identified proteins were annotated with 1747 GO terms, of which 165, 280, and 1302 belonged to the category of cellular component (CC), biological process (BP), and molecular function (MF), respectively (Table S2). The top 10 GO terms in CC, BP, and MF are presented in Figure 2A. The “oxidation-reduction process” is the GO term containing the most proteins in BP, indicating that this process likely plays a crucial role in muscle function. Moreover, most proteins were annotated as “protein binding” in molecular function. We then performed an additional KEGG annotation of these proteins, which categorized them into 33 pathways (Figure 2B and Table S2). The largest numbers of proteins were annotated as “signal transduction” and “global and overview maps” pathways, with protein numbers of 296 and 382, respectively (Figure 2B). Overall, 1433 proteins were annotated into 24 categories in COG (Table S2). Most proteins were annotated as involved in “posttranslational modification, protein turnover, chaperones,” “translation, ribosomal structure and biogenesis,” “general function prediction only,” and “signal transduction mechanisms” (Figure 2C). IPR analysis data showed that these proteins were classified into 1748 protein domains (Table S2). The top 20 protein domains are shown in Figure 2D, and these proteins were primarily annotated as “RNA recognition motif domain” and “protein kinase domain” (Figure 2D).

### 3.3. Analysis of Differentially Expressed Proteins (DEPs) between BF and SOL Muscles

**Figure 3:**
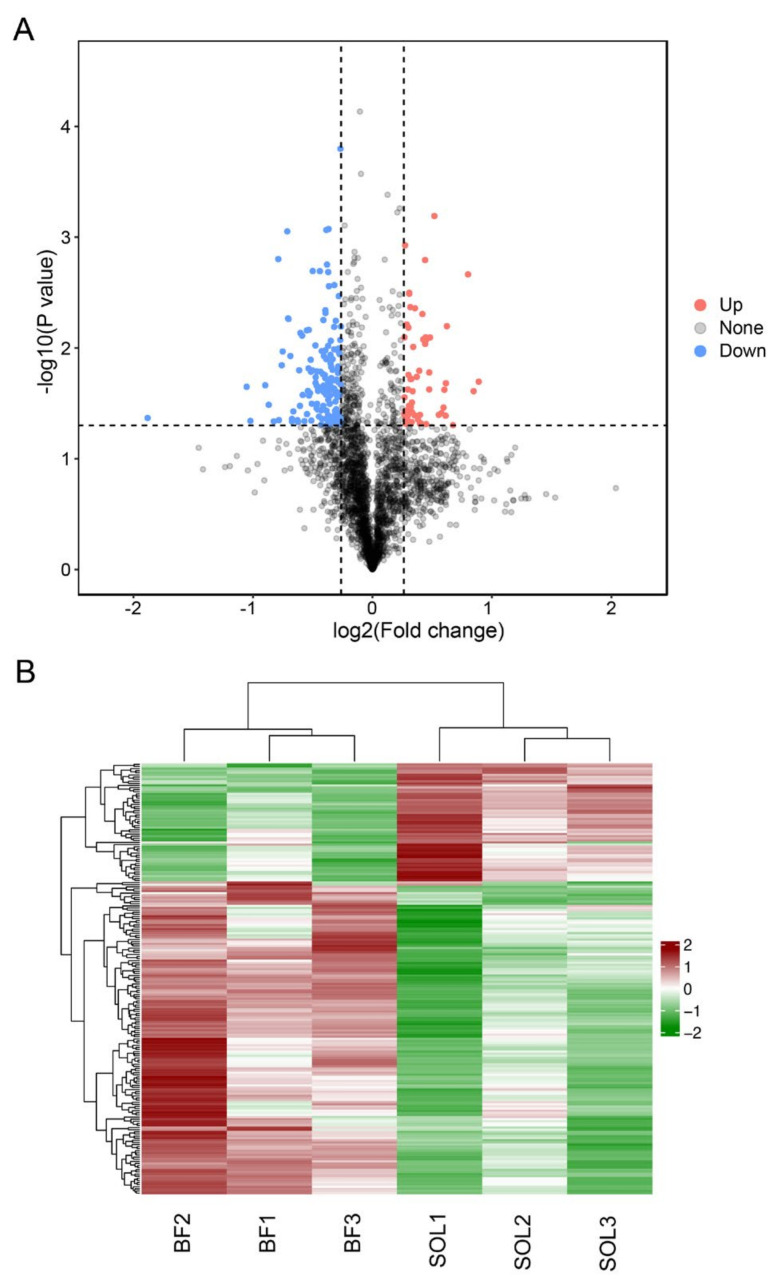
Analysis of DEPs (differentially expressed proteins) among BF and SOL muscles. (**A**) Comparing the differences in proteins between BF and SOL using a volcano plot. The X–axis displays the log2(Fold change) value, while the Y–axis shows the −log10(*p* value). Red, blue, and black represent proteins that are up-regulated, down-regulated, and show no difference, respectively. (**B**) Heatmap displaying the abundance of DEPs expression in the BF and SOL tissue samples. The expression abundance of each protein is colored from red (high intensity) to green (low intensity) using a z–scored log2(Fold change) value.

We conducted a differential expression analysis of proteins between the glycolytic BF and oxidative SOL muscles to identify proteins that may be involved in the presence of variations in muscle fiber type. Between BF and SOL, 204 DEPs were found, of which 148 were down-regulated and 56 were up-regulated in the SOL muscle (Figure 3A). Detailed information on the DEPs is provided in Table S3. The top five up-regulated DEPs were carbonic anhydrase 3 (CA3), myoglobin (MB), myosin-7 (MYH7), cytochrome c domain–containing protein (LOC1525869), and histone H2A (H2AC2), while the top 5 down-regulated DEPs were tumor-suppressor candidate 5 (TUSC5), myosin-4 (MYH4), perilipin, protein containing the fibrillar collagen NC1 domain, and the collagen type-I α 1 chain (COL1A1) (Table S3). We carried out a cluster analysis of the levels of protein expression in the six muscle samples to better understand the expression patterns of DEPs across various muscle samples. The cluster heatmap revealed that the up-regulated and down-regulated DEPs were divided into two categories. (Figure 3B). These findings imply that the change in muscle fiber type is linked to the DEPs.

### 3.4. Functional Enrichment Analysis of DEPs

**Figure 4:**
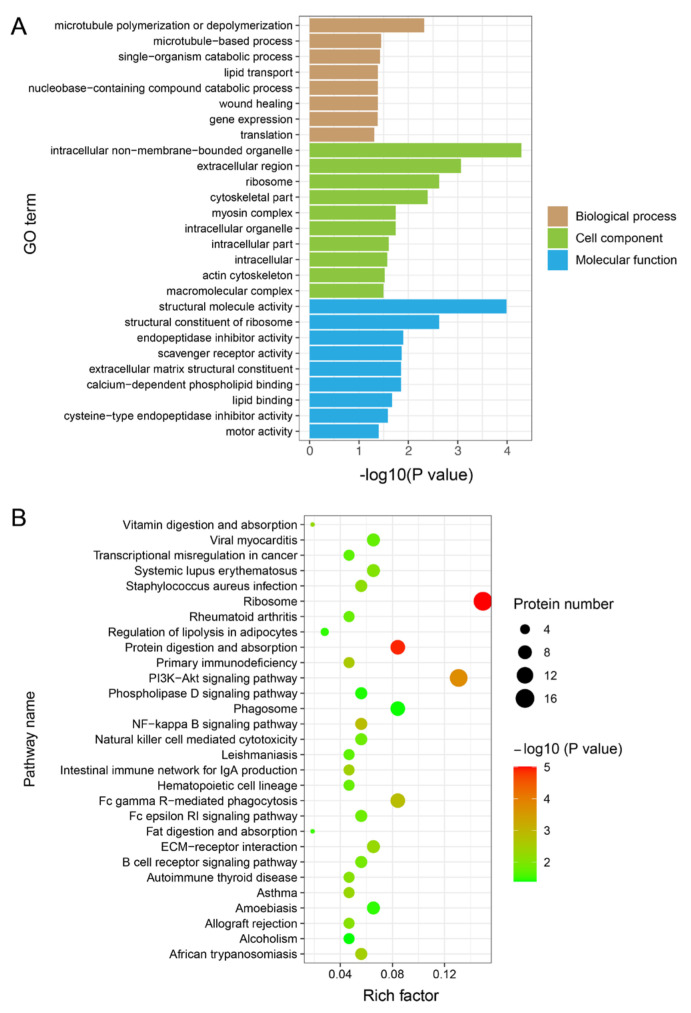
GO and KEGG functional enrichment analyses of the DEPs. (**A**) GO enrichment analysis of DEPs. The X–axis represents the −log10(*p* value), whereas the name of the GO term is presented on the Y–axis. (**B**) KEGG enrichment analysis of the DEPs. The X–axis displays the rich factor, and the Y–axis represents the pathway name. The number of proteins and the −log10(*p* value) are indicated by the size and color of the dots, respectively.

We carried out GO and KEGG enrichment analyses based on DEPs to better comprehend the biological roles of the DEPs among various muscle types. The GO analysis results revealed that 27 GO terms were significantly enriched, with eight GO terms for BP, ten for CC, and nine for MF (Figure 4A and Table S4). For example, GO terms “intracellular non-membrane-bound organelle,” “cytoskeletal part,” and “myosin complex” were enriched in CC. The term “cytoskeletal part” could be expected because it is a key pathway involved in glycolysis, as demonstrated in a previous study [32]. The KEGG enrichment analysis showed that these proteins were enriched significantly in 29 pathways, including “ribosome,” “protein digestion and absorption,” “PI3K-Akt signaling pathway,” and “NF-kappa B signaling pathway” (Figure 4B and Table S5). The PI3k-Akt signaling pathway was shown to be involved in glycolysis in a previous study [33] (Figure 4B and Table S5).

### 3.5. Analysis of Protein–Protein Interaction (PPI) in the DEPs

**Figure 5:**
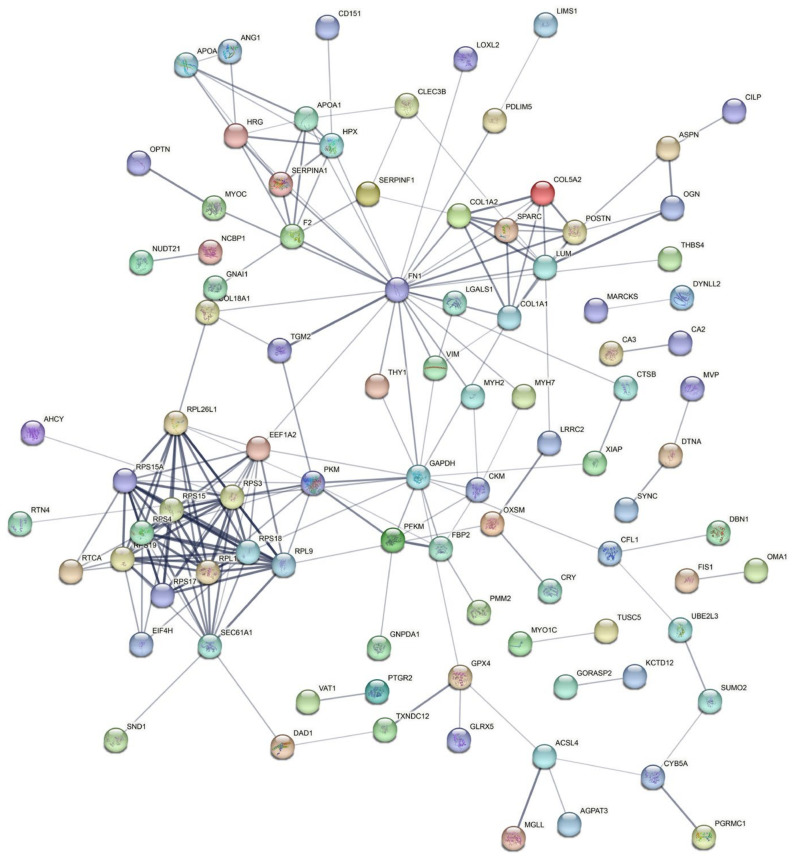
Protein–protein interaction (PPI) regulatory network analysis of the DEPs. The lines represent proteins that interact with each other.

To understand the regulation of muscle fiber types via interactions between DEPs, we analyzed PPIs among the DEPs using the STRING database. In the PPI regulation network constructed, a total of 93 proteins were predicted to participate in 207 interactions (Figure 5 and Table S6). Specifically, CA3 (carbonic anhydrase 3) was predicted to play a crucial function in the regulatory network by its interaction with the CA2 (carbonic anhydrase 2) proteins, which regulate muscle fiber types. PKM, a glycolytic key enzyme, can interact with RPL26L1, RPS4, RPS15A, RPL9, RPL11, RPS15, RPS3, and TGM2 proteins.

## 4. Discussion

It has been established that one of the major elements determining meat quality is the difference in skeletal muscle fiber types [6,34]. An increased percentage of type IIb muscle fiber is more likely to produce PSE meat by lowering the pH [35]. Oxidative muscles have better meat quality than glycolytic muscles [16]. Therefore, identifying mechanisms that allow conversion between muscle fiber types is crucial for the improvement in meat quality. In recent years, TMT-labeled quantitative proteomics has been widely applied to study protein expression profiles related to meat quality [19,25,36]. We identified a total of 2667 proteins from samples of slow/oxidative SOL muscle and fast/glycolytic BF muscle in the current study using TMT-labeled quantitative proteomics. These data have the potential to provide information useful for future studies on muscle development and transformation of muscle fiber types in pigs. Furthermore, we identified 204 DEPs between the BF and SOL muscle samples that are potential key proteins in the regulation of muscle fiber type conversion. For instance, MYH7 and MYH4 proteins, which are recognized as marker proteins for slow- and fast-type muscle fiber types, respectively, were shown to be up-regulated and down-regulated in SOL muscle. This observation indicates that the differences in protein abundance identified in this study are reliable. Interestingly, TNNT3, a fast sarcomeric myosin gene, was significantly down-regulated in SOL muscle, and a previous study reported that this protein regulates fast sarcomeric unit formation [37] and skeletal muscle contraction [38]. This observation suggests that TNNT3 is a key protein in promoting fast-type muscle formation in pigs. We also observed that carbonic anhydrase 3 (CA3) is significantly up-regulated in SOL muscle. CA3 is present abundantly in slow–type I fibers and facilitates the conversion of fast- to slow-type muscles [39,40]. These data suggest that some of the DEPs identified here influence pork quality by regulating muscle fiber type conversion.

GO enrichment analysis of the DEPs revealed the involvement of some GO terms in the development of muscle fiber types, such as actin cytoskeleton, myosin complex, and the cytoskeletal part. DEPs MYH4, MYO1C, TNNT3, MYH2, and MYH7 were enriched in the actin cytoskeleton GO term. Slow muscle fibers contract slowly and are resistant to fatigue, whereas fast muscle fibers contract more rapidly and are easily fatigued [41,42]. Previous evidence suggests that the contractile function of muscle fibers is regulated by the interaction of actin and myosin filaments [3]. Our results suggest that DEPs impact the contractile properties of the muscle fibers by influencing the binding of actin and myosin. Several signaling pathways associated with different types of muscle fibers were identified using KEGG enrichment analysis of the DEPs, which include the PI3K-Akt and NF-kappa B signaling pathways. A recent study demonstrated that the PI3K/AKT/FOXOs pathway plays a crucial function in the maintenance of muscle fiber types [43]. Skeletal muscle fiber size and its defense against disease-induced muscle atrophy are regulated by the activation of the Akt/mTOR pathway [44]. Thus, our results suggest that the DEPs regulate porcine muscle fiber types by involving the PI3K-Akt signaling pathway. Lack of NF-κB can lead to a reduction in mitochondrial content and function, which ultimately affects the oxidative metabolic processes in the muscle through PGC-1β [45]. Our results imply that NF-κB mediates a process by which DEPs affect the transformation of porcine muscle fibers. However, the specific mechanisms for DEPs regulating porcine muscle fiber phenotypes through these signaling pathways need to be confirmed by future studies.

**Figure 6:**
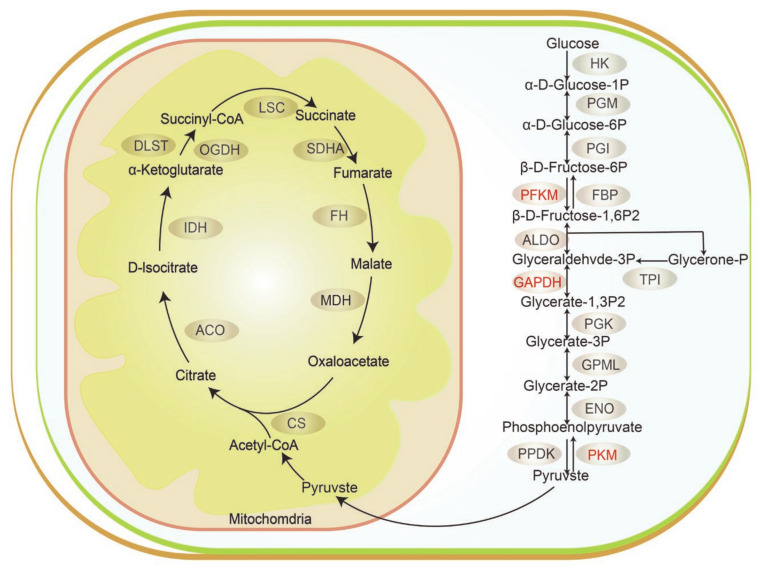
DEPs are involved in the glycolytic process. The three down-regulated DEPs (PFKM, GAPDH, and PKM) are labeled in red.

There are different metabolic properties across slow- and fast-type muscle fibers, with the former being determined by mitochondrial oxidative enzymes and the latter by glycolytic enzymes [46]. Notably, some of the glycolytic enzymes, PFKM, GAPDH, and PKM, were significantly more abundant in the fast-type muscle samples used in this study (Figure 6). PFKM is the enzyme that converts fructose-6-phosphate into fructose-1,6-bisphosphate by phosphorylation, resulting in the entry of glucose into glycolysis. It has been demonstrated previously that MyHC I and MyHC IIa protein expression levels were significantly elevated in the skeletal muscles of PFKM-deficient mice [47]. PKM is the last key enzyme in glycolysis that catalyzes the conversion of phosphoenolpyruvate to pyruvate [48]. PKM is prominently expressed in glycolytic skeletal muscle (longissimus thoracis), and its abundance is associated with pork quality in terms of pH and drip loss [16]. Additionally, GADPH also affects meat quality by reducing the pH of muscle after slaughter [49]. Our studies suggest that these three DEPs act as key enzymes in the glycolytic process and affect the glycolytic muscle phenotype.

The PPI regulatory network constructed based on the DEPs identified in our study revealed that DEPs affect muscle fiber type conversion through the interactions between these proteins. CA3 is a chief candidate protein involved in the promotion of slow-type muscle development [40]. Our PPI regulatory network suggests that CA3 and CA2 directly interact with each other, demonstrating that they collaborate to influence the phenotype of slow-type muscle. In addition, several key glycolytic enzymes, including PFKM, GAPDH, and PKM, potentially interact directly with other DEPs. Interestingly, PFKM, GAPDH, and PKM are reported to interact with FBP2, which is the key enzyme for the synthesis of fructose-6-phosphate from fructose-1,6-bisphosphate [50]. It was previously demonstrated that increasing FBP2 activity led to elevated glycolytic flux in the extensor digitorum longus (EDL) muscle [50]. Thus, FBP2 may facilitate slow-to-fast muscle conversion by interacting with PFKM, GAPDH, and PKM. These findings offer resources for future investigations on the regulatory network governing the change in muscle fiber types in pigs.

## 5. Conclusions

In summary, we performed proteome analyses and identified 204 DEPs between the BF and SOL muscles of pigs. The DEPs were found to be primarily enriched in GO terms and signaling pathways associated with muscle fiber types. We constructed a PPI regulatory network that illustrates the roles of glycolytic and oxidative proteins in muscle type variation in pigs. Moreover, these results need to be further confirmed in larger populations. These findings offer novel insights into the regulatory mechanisms governing the muscle fiber types and meat quality in pigs.

## Data Availability

All mass spectrometry data used in this study have been submitted to the iProX database (accession number IPX0005498000).

## Supplementary Materials

The following supporting information can be downloaded at: https://www.mdpi.com/article/10.3390/genes14020361/s1, Table S1: The identified proteins in this study; Table S2: Functional annotation of identified proteins; Table S3: Information of DEPs; Table S4: GO enrichment analysis of DEPs; Table S5: KEGG enrichment analysis of DEPs; Table S6: PPI regulatory network of DEPs.
